# Dexmedetomidine Protects Against Septic Liver Injury by Enhancing Autophagy Through Activation of the AMPK/SIRT1 Signaling Pathway

**DOI:** 10.3389/fphar.2021.658677

**Published:** 2021-04-26

**Authors:** Qing Yu, Liying Zou, Xiu Yuan, Fang Fang, Feng Xu

**Affiliations:** ^1^ Department of Intensive Care Unit, Children's Hospital of Chongqing Medical University, Chongqing, China; ^2^ Ministry of Education Key Laboratory of Child Development and Disorders, National Clinical Research Center for Child Health and Disorders, China International Science and Technology Cooperation Base of Child Development and Critical Disorders, Children's Hospital of Chongqing Medical University, Chongqing, China; ^3^ Chongqing Key Laboratory of Pediatrics, Chongqing, China

**Keywords:** sepsis, liver injury, dexmedetomidine, autophagy, clp

## Abstract

**Background:** Liver injury is one of the serious complications of sepsis. Previous studies suggested that dexmedetomidine (DEX) could alleviate cecal ligation and puncture (CLP)-induced liver injury. However, it is unclear whether the protective effect of DEX on sepsis-induced liver injury is related to autophagy.

**Methods:** Mice (*n* = 105) were randomly divided into the following groups: (i) CON group (Sham); (ii) CLP group (CLP-induced liver injury + saline); (iii) CLP + DEX group (CLP-induced liver injury + DEX). Mouse models of sepsis-induced liver injury were established using CLP. DEX or normal saline was administered by intraperitoneal injection at 0, 2, and 4 h after CLP surgery. The mortality rate within 120 h was calculated. The levels of alanine aminotransferase (ALT), aspartate aminotransferase (AST), and inflammatory cytokines were measured at 6, 12, and 24 h in each group. Hematoxylin and eosin staining assay was carried out to detect the morphological changes of mouse liver cells in each group. The levels of autophagy-associated proteins LC3II, Beclin-1, p62, and LAMP-2 were detected in three groups of mice using western blotting. The expression of LC3II was detected using immunofluorescence. Transmission electron microscopy (TEM) of liver tissue was used to observe autophagosomes and autophagosome–lysosomes. Lastly, the effect of DEX on the AMPK/SIRT1 pathway-associated protein levels were detected using western blotting. Meanwhile, we used L0-2 cells infected with mRFP-GFP-LC3 adenovirus to further analyze the role of SIRT1 in DEX-induced autophagy in liver injury model *in vitro*.

**Results:** DEX significantly improved the survival rate of septic mice at the early stage and ameliorated the pathology of sepsis-induced liver injury. The level of autophagy-associated proteins, phosphorylated (*p*)-AMPK/AMPK, and SIRT1 in the liver of CLP-induced sepsis mice peaked at 12 h post-CLP and decreased significantly at 24 h. In the CLP + DEX group, the levels of autophagy-associated proteins, *p*-AMPK/AMPK, and SIRT1 increased, whereas inflammatory cytokines decreased at 24 h. The autophagosome structure was clearly observed at different time points in the CLP + DEX group. In the *in vitro* hepatocyte injury model, the SIRT1 inhibitor significantly increased intracellular ROS levels and reversed the effect of DEX on autophagy flux.

**Conclusion:** We demonstrated a novel mechanism in which DEX protects against CLP-induced liver injury. DEX enhances autophagy, which alleviates the inflammatory responses in CLP-induced liver injury by regulating the SIRT1/AMPK pathway.

## Introduction

Sepsis is a life-threatening disease caused by the host’s unbalanced response to infection, which can lead to severe systemic inflammation and multiple organ dysfunction syndromes (Selim et al., 2019). Liver dysfunction is one of the clinical complications closely related to sepsis, and has been used as a strong predictor of high mortality and poor prognosis (Kramer et al., 2007). Clinical data show that the incidence of sepsis-associated liver dysfunction ranges from 34 to 46% (Yan et al., 2014). Once patients with sepsis develop liver dysfunction, the mortality rate increases by nearly 54% compared with other organ complications (Yan et al., 2014).

Sedation and analgesia are the basic treatment measures for patients with sepsis, especially those undergoing mechanical ventilation. For patients with sepsis, evidence has shown that most sedatives have anti-inflammatory effects and may increase susceptibility to infection (Nseir et al., 2010). However, the α_2_-adrenoceptor agonist is the one possible exception to this observation (Sanders et al., 2011). Dexmedetomidine (DEX), a highly selective α_2_-adrenoreceptor agonist, was reported to be beneficial for sepsis in a number of animal and clinical studies Tasdogan et al. (2009), Pandharipande et al. (2010), Chen et al. (2015), Jin et al. (2019), Ohta et al. (2020); however, its protective mechanism is not completely understood.

Autophagy is a lysosome-dependent process of removing damaged proteins and organelles from cells (Mizushima and Levine, 2010). Currently, autophagy is recognized as an effective target to reverse immunosuppression compromised by sepsis (Ren et al., 2017). Several studies have shown that inadequate or inhibited autophagy is associated with mitochondrial dysfunction, liver injury/failure, and adverse outcomes (Vanhorebeek et al., 2011; Gunst et al., 2013). Dexmedetomidine was reported as beneficial for septic challenge via inducing autophagy. Previous studies demonstrated that DEX, by regulating the autophagy level, exerts an organ-protective effect against sepsis, including septic acute kidney injury Yang et al. (2020), Zhao et al. (2020) and septic myocardial dysfunction (Yu et al., 2019). However, whether the protective effect of DEX on sepsis-induced liver injury is related to autophagy remains unclear.

Adenosine monophosphate-activated protein kinase (AMPK) is a crucial kinase involved in the regulation of energy metabolism, which plays a protective role in the acute stage of liver injury (Yu et al., 2019). Sirtuin 1 (SIRT1), a nicotinamide adenine dinucleotide (NAD+)-dependent histone deacetylase, is a major regulator of energy metabolism and an important part of cell survival, such as cell apoptosis, proliferation, and inflammation (Salminen and Kaarniranta, 2012). Studies have confirmed that DEX attenuates sepsis induced by acute lung injury through the SIRT1-dependent AMPK pathway (Wang et al., 2020). Activation of AMPK signaling is effective in upregulating autophagy (Ho et al., 2016). In addition, AMPK provides NAD + for the activity of SIRT1, thereby establishing a positive feedback loop, which is expected to result in prolonged autophagy (Canto et al., 2009). A number of studies have confirmed the anti-inflammatory and organ protective effects of DEX, suggesting that its protective mechanism might be related to the autophagy pathway. However, there is no specific study showing how autophagy changes in a sepsis model treated with DEX at different time points. Therefore, in the present study, we aimed to observe the time-course of changes in autophagy and liver function in septic mice treated with DEX. Furthermore, we aimed to verify whether the hepatoprotective mechanisms of DEX were associated with regulation of the SIRT1/AMPK pathway.

## Materials and Methods

### Animals, Cecal Ligation and Puncture Surgery, and Treatment

All experiments were approved by the Animal Care Committee of the Chongqing Medical University. One hundred and five male C57BL/6 mice, weighing 18–22 g and aged 6–8 weeks old (Chongqing Medical University, Chongqing, China) were housed under a 12-h light/12-h dark cycle at a constant temperature (22°C) with free access to food and water. After 1 week of acclimation, all mice were randomly divided into the following groups: (i) CON group (Sham surgery, *n* = 13); 2) Cecal ligation and puncture (CLP) group (CLP-induced liver injury + saline, *n* = 46); (iii) CLP + DEX group (CLP-induced liver injury + dexmedetomidine, *n* = 46).

All mice were anesthetized using an intraperitoneal injection of pentobarbital sodium 50 mg/kg. Sepsis was induced by CLP as described previously (Rittirsch et al., 2009). After the abdominal wall was sutured, 1 ml of 0.9% sodium chloride solution was injected subcutaneously for fluid resuscitation. Each animal completed CLP in about 10 min. A laparotomy and bowel operation without ligation and puncture were performed on the mice in the CON (sham) group. DEX (20 μg/kg, Nhwa Pharmaceutical Co., Ltd. Jiangsu, China) or normal saline was administered by intraperitoneal injection at 0, 2, and 4 h after CLP surgery. The liver and serum were obtained 6, 12, and 24 h after CLP-induced sepsis for further studies.

### Mortality Rate

Seventy mice were randomly divided into three groups (CON, *n* = 8; CLP, *n* = 31; CLP + DEX, *n* = 31). The mice were subjected to CLP or sham surgery and administered as stated in *Mortality Rate*. All the animals were observed after surgery and administration. Observation was performed every 12 h for the first 2 days and every 24 h for the following 3 days. The time when an animal died was recorded. The animals were observed up to 120 h after surgery and then sacrificed using carbon dioxide asphyxia. The mortality rate within 120 h was calculated.

### Assessment of Liver Function and Enzyme-Linked Immunosorbent Assay of Inflammatory Cytokines

Blood samples were collected from the orbital sinus at different points after CLP. After blood coagulation for 30 min, serum was collected by centrifugation at 3,000 rpm for 10 min at ambient temperature. Serum levels of alanine aminotransferase (ALT) and aspartate aminotransferase (AST) were determined using commercial kits (Neobioscience, Jiancheng Bioengineering Institute, Nanjing, China).

Levels of inflammatory cytokines, including tumor necrosis factor alpha (TNF-*α*), interleukin (IL)-1, IL-6, and IL-10 were measured using an ELISA kit (Neobioscience). The optical density (OD) of each well was read at 450 nm and the concentrations of inflammatory cytokines were determined quantitatively according to a standard curve.

### Hematoxylin-Eosin Staining Assay and Ultrastructural Observations

A portion of liver tissue (left lateral liver lobe) obtained from each group was placed in 4% paraformaldehyde for more than 24 h, dehydrated, and embedded in paraffin wax. Next, the liver tissues were sectioned at 5 μm and fixed on a glass slide. The sections were stained with H&E. Hematoxylin was incubated with the sections for 4 min, followed by eosin for 90 s. Histological analyses were evaluated at × 400 magnification using a Leica DM 2700 P microscope (Leica Microsystems GmbH, Wetzlar, Germany).

Another part of the liver (right lateral liver lobe) from each group was fixed overnight with 2.5% glutaraldehyde, and then washed three times with 0.1 M phosphate-buffered saline (PBS) for 15 min each time. Tissues were then fixed with 1% osmium for 1 h and dehydrated with different concentrations of alcohol (50, 70, 80, 90, and 95%) for 15 min. The tissues were then placed in epoxy resin and polymerized at 60°C for 2 h. Finally, ultrathin sections (60 nm) were acquired, which were doubled stained with uranyl acetate and lead citrate. The sections were observed on a transmission electron microscope (Hitachi HT7700, Tokyo, Japan)

### Immunofluorescence Staining of LC3II and Immunohistochemistry Analysis of Sirtuin1

Immunofluorescent staining was performed on paraffin vertical sections using antibodies against lipid-modified microtubule-associated proteins 1 A/1 B light chain 3 B (LC3II). Briefly, the liver sections were dewaxed in xylene, rehydrated, and heated in a microwave with citrate buffer (pH  6.0) for antigen retrieval before exposure to the primary antibody. Then, sections were immersed in 3% hydrogen peroxide for 10 min to block endogenous peroxidase. Nonspecific immunoreactions were blocked using 5% inactivated goat serum in PBS for 30 min at room temperature. The sections were incubated with anti-LC3II (1:100, Cell Signaling Technology (CST), Danvers, MA, United States) antibodies overnight at 4°C. Sections were washed with PBS extensively and incubated with the corresponding Alexa Fluor 555-conjugated secondary antibody (1:1,000; Beyotime Institute of Biotechnology, Jiangsu, China) and 4ʹ,6-diamidino-2-phenylindole (DAPI) (5 μg/ml, Beyotime Institute of Biotechnology) at room temperature for 1 h in a dark box. The sections were washed with PBS three times again and then sealed with coverslips. Fluorescence images were acquired using a Nikon Eclipse Ni inverted microscope (TE 2000; Nikon, Tokyo, Japan).

Immunohistochemical staining was performed on paraffin vertical sections using antibodies against SIRT1. Briefly, the liver slides were deparaffinized in xylene and the antigen was retrieved by microwaving. After blocking in 5% normal goat serum and permeabilized in 0.5% Triton X-100 in PBS, the slices were incubated with anti-SIRT1 antibodies (Proteintech, Rosemont, IL, United States; 13161-1-AP, 1:100) at 4°C overnight. The sides were then incubated with the corresponding secondary IgG/HRP polymer antibodies (Polink-1 HRP DAB Detection System, ZSGB-BIO, Beijing, China). Negative controls were subjected to the same procedures but without the primary antibody. The stained sections were examined under a Nikon Eclipse microscope (55i; Nikon, Tokyo, Japan). Protein expression was evaluated as the dark brown staining in the whole section, and the mean optical density was calculated using Image pro plus software version 6.0 (Media Cybernetics, Rockville, MD, United States).

### Cell Culture and Treatments

Normal human hepatic cell line of L-02, purchased from the Cell Bank of Chinese Academy of Sciences (Shanghai, China), was cultured in Roswell Park Memorial Institute (RPMI) 1,640 medium (Gibco, Grand Island, NY, United States) containing 10% fetal bovine serum (Gibco), penicillin of 100 U/ml, and streptomycin of 100 μg/ml in a humidified incubator, with CO_2_ at 5% and 37°C. The dose of the d-galactosamine (GalN)/lipopolysaccharide (LPS) model (D-GalN/LPS: 10 mM/10 ng/ml) was determined based on previous research Liu et al. (2018) and our pre-experimental results. LPS (055: B5) and D-GalN (G1639) were obtained from Sigma-Aldrich Chemical Company (St. Louis MO, United States). In subsequent experiments, L-02 hepatocytes were divided into four groups: (a) Control group: Cells were cultured in RPMI 1640 medium alone. (b) D-GalN/LPS group: Cells were stimulated by D-GalN (10 mM)/LPS (10 ng/ml). (c) DEX + D-GalN/LPS groups: Cells were incubated with DEX (1, 10 μM) and D-GalN (10 mM)/LPS (10 ng/ml). (d) DEX + EX527+D-GalN/LPS groups: Cells were incubated with DEX (1, 10 μM), EX527 (10 μM), and D-GalN (10 mM)/LPS (10 ng/ml). According to the results of Cell Counting Kit-8 (CCK-8) assays (MedChemExpress, Monmouth Junction, NJ, United States), the concentration of DEX treatment in the subsequent experiments was determined as 1 μM.

### Measurement of Intracellular ROS Production

The intracellular ROS level was evaluated using the 2ʹ,7ʹ-dichlorodihy-drofluorescein diacetate (DCFH-DA) method. Cells (8 × 10^4^ cells/mL) were seeded into 12-well plates and cultured for 24 and 48 h. After being washed with PBS, the cells were treated with 10 μM DCFH-DA for 30 min at 37°C in a humidified incubator. After removing the redundant DCFH-DA, the cells were carefully washed with PBS three times. Representative images were captured using an inverted fluorescence microscope (TE 2000; Nikon) with excitation wavelength of 488 nm and an emission wavelength of 530 nm. The fluorescence intensity reflecting ROS contents were analyzed using IPP software (Version 6.0, Media Cybernetics).

### Double-Labeled Adenovirus mRFP-GFP-LC3 Transfection and Autophagy Detection

The L-02 hepatocytes were seeded and cultured in 12-well plates for 48 h, and then mRFP-GFP-LC3 adenovirus (GV540, Genechem, Shanghai, China) was transfected according to the manufacturer’s protocol. The cells were then divided into four groups: Control group; D-GaIN/LPS group; DEX + D-GalN/LPS group, and DEX + EX527+D-GalN/LPS group. After 24 and 48 h of cell culture, the formation of autolysosomes was detected and analyzed using an inverted fluorescence microscope (TE 2000; Nikon), and the cell were photographed under × 200 magnification. On the images, yellow spots and red spots refer to autophagosomes and autolysosomes, respectively.

### Western Blotting Analysis

Liver tissue was homogenized in Radioimmunoprecipitation assay lysis buffer (Beyotime Institute of Biotechnology) with protease inhibitors by sonication. The proteins were quantified using a bicinchoninic acid assay. Total lysate (60 µg protein/lane) was separated using 10–12% sodium dodecyl sulfate-polyacrylamide gel electrophoresis and the separated proteins were transferred to polyvinylidene difluoride (PVDF) membranes. The PVDF membranes were then blocked with 5% non-fat milk before incubation with primary antibodies against LC3II (CST; #2775, 1:1,000), Beclin-1 (CST, #3738, 1:1,000), p62 (also known as Sequestosome 1) (GeneTex, Irvine, CA, United States of America; GTX100685, 1:1,000), lysosomal associated membrane protein 2 (LAMP-2) (Abways, Shanghai, China; CY5518, 1:1,000), AMPK (CST, #5832, 1:1,000), *p*-AMPK (CST, #2535, 1:1,000), SIRT1 (CST, #9475, 1:1,000), and GAPDH (GeneTex, GTX100118, 1:5,000) overnight at 4°C. The membranes were washed three times for 5 min each and then incubated with appropriate HRP-conjugated secondary antibodies (goat anti-rabbit; Gengtex, GTX213110–01, 1:7,000) at room temperature for 1 h. Immunoreactive protein bands were visualized using an ECL Western Blotting Detection reagent (Millipore, Billerica, MA, United States). The protein bands were then detected and quantified using a Bio-Rad imaging system (Bio-Rad Laboratories, Inc. Hercules, CA, United States).

### Statistical Analysis

The results are presented as the mean ± standard deviation (SD). One-way analysis of variance followed by Tukey’s multiple comparison test were used for statistical analysis to compare values among all groups. The mortality rates among groups were compared using the Kaplan-Meier method. Graphs were made using GraphPad Prism5 (GraphPad Inc. San Diego, CA, United States of America). SPSS software package version 22.0 (IBM Corp. Armonk, NY, United States) was used for data analysis. Statistical significance was defined as *p* < 0.05.

## Results

### Dexmedetomidine Significantly Improved the Survival Rate of Septic Mice at the Early Stage

**FIGURE 1:**
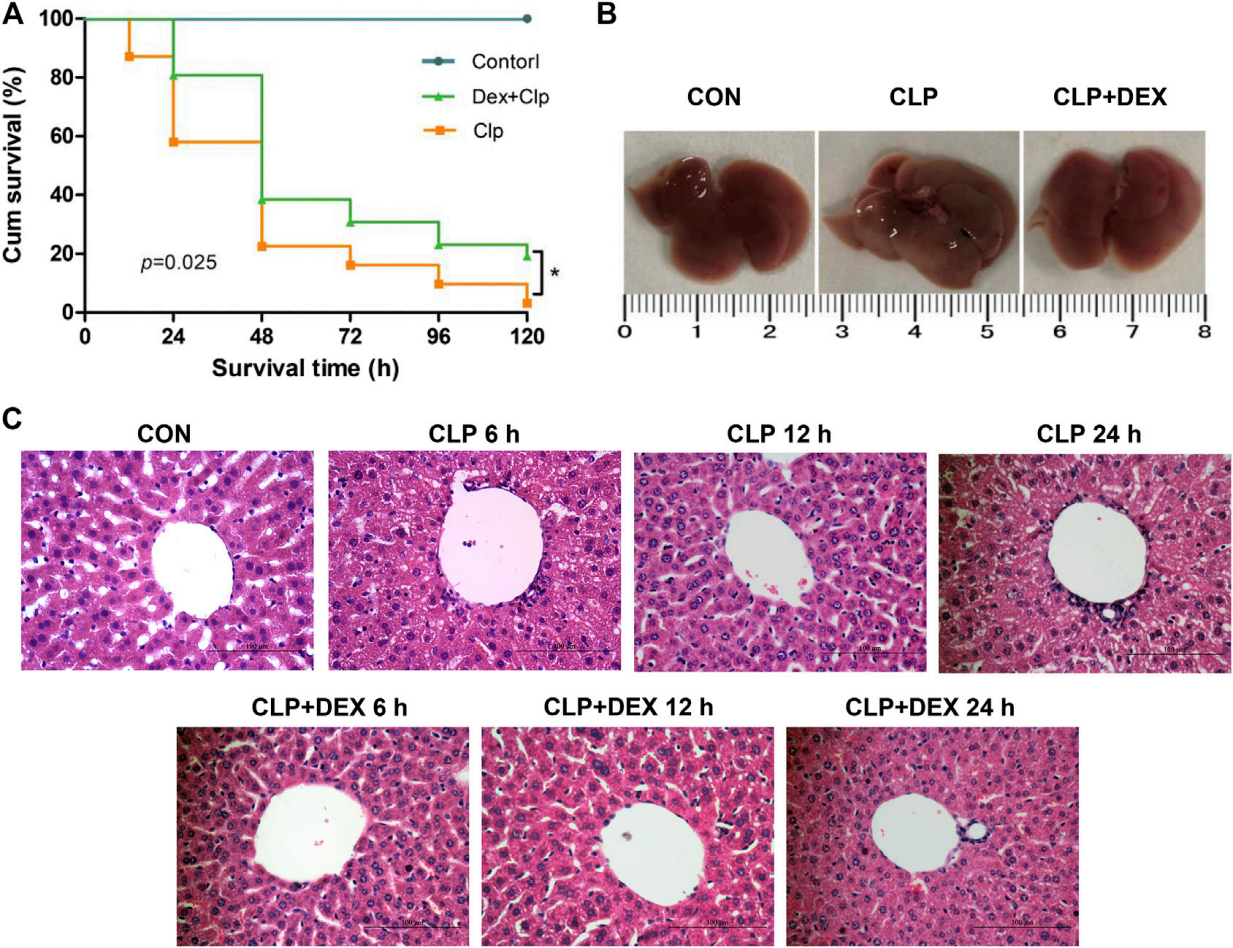
Protective effect of DEX in CLP-induced liver injury. **(A)** Effect of DEX on the survival rate of septic mice in different groups. C57 mice were subjected to CLP surgery, and survival (*n* = 31 for each CLP group, and *n* = 8 for control group) was monitored for 120 h. **(B)** Image of entire liver taken from mice 24 h after CLP surgery in the CON, CLP, and CLP + DEX groups. **(C)** The role of DEX in pathological liver injury in septic mice in CON, CLP, and CLP + DEX group at 6, 12, and 24 h (Hematoxylin-and-Eosin stain; magnification: × 400). *p < 0.05. CON: control; CLP: cecal ligation and puncture; DEX: dexmedetomidine.

As shown in Figure 1A, dexmedetomidine significantly improved the survival rate of mice in the early stage of sepsis. The survival rates of the CLP group and CLP + DEX group were 81 and 100% at 12 h, 58.1 and 83.8% at 24 h, and 38.7 and 61.3%, respectively, at 36 h. The 5-day survival rate of the mice in the CLP group was 3.2% and that of the CLP + DEX group was 12.9%. The difference in the 5-day survival rate of the CLP group and CLP + DEX group was statistically significant (*p* = 0.025). Sham-operated mice in the CON group had a 100% survival rate.

### Dexmedetomidine Ameliorated the Pathology of Sepsis-Induced Liver Injury

Histopathological changes are direct signs of liver injury. The liver tissue of mice was collected at 6, 12, and 24 h after CLP surgery. The hepatocytes in the liver tissue of CON group were neatly arranged, with no edema and no cell necrosis. However, compared with the CON group, obvious pathological changes, such as inflammatory cell infiltration, hepatocyte swelling, disordered arrangement, and cell necrosis were observed in the liver tissues of the CLP group at 6 and 24 h. The pathological changes of the liver tissue in CLP + DEX group were improved compared with those of the CLP group at different time points (Figure 1C). The whole liver tissue in the CLP group was more swollen than that in the CON group and CLP + DEX group at 24 h after CLP surgery (Figure 1B).

### Dexmedetomidine Ameliorated Early Liver Dysfunction in Septic Mice

**FIGURE 2:**
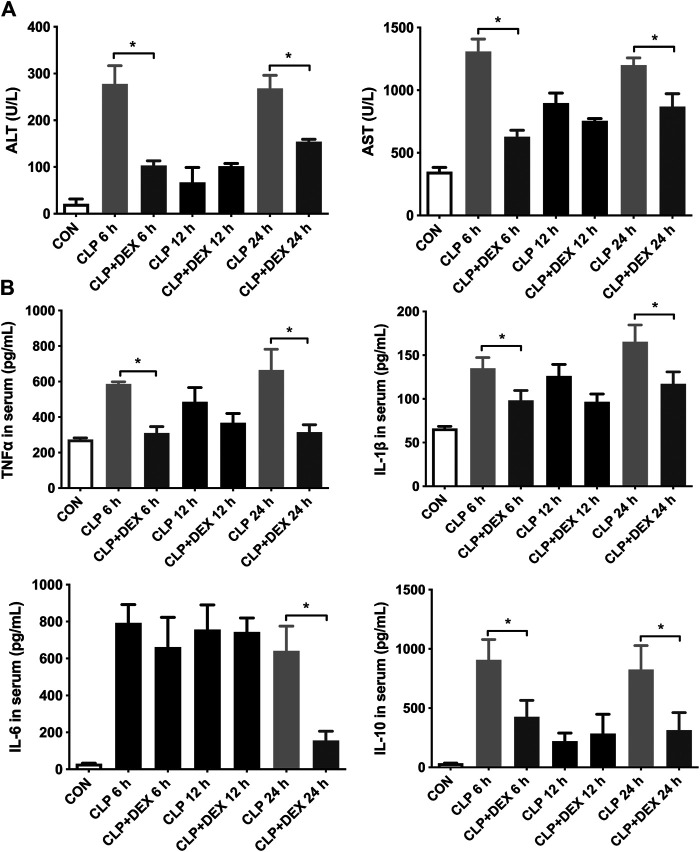
DEX reduced liver dysfunction and inflammation in CLP-induced liver injury. **(A)** DEX improved liver dysfunction in CLP-induced liver injury: The levels of serum ALT and serum AST at 6, 12, and 24 h in mice. **(B)** The content of inflammatory cytokines in serum, including TNF-α, IL-1β, IL-6, and IL-10, as determined using ELISA. *p < 0.05.

The results showed that the ALT and AST levels increased significantly in the CLP group at 6 h, decreased at 12 h, and then increased significantly at 24 h. In contrast, the levels of ALT and AST in the CLP + DEX group were significantly lower than those in CLP group at 6 and 24 h (*p* < 0.05). There was no significant difference between the two groups at 12 h (Figure 2A). These findings suggested that DEX could reduce the level of liver injury indicators in the first 24 h of sepsis, and the severity of liver injury in the CLP group was relatively mild at 12 h.

### Dexmedetomidine Decreased the Levels of Inflammatory Cytokines in Septic Mice

To verify the anti-inflammatory effect of DEX, we examined changes in the serum levels of inflammatory cytokines. The ELISA results revealed that CLP induced an increase in the levels of cytokines in mice. As shown in Figure 2B, compared with those in the CLP + DEX group, the levels of TNF-*α*, IL-1β, and IL-10 increased significantly in the CLP group at 6 h after sepsis induction. At 12 h after sepsis induction, the levels of TNF-*α*, IL-1β, and IL-10 decreased in the CLP group, and no significant difference was found between the CLP group and the DEX + CLP group. Up to 24 h, the levels of the three above-mentioned inflammatory cytokines in the CLP group were significantly higher than those in the CLP + DEX group. This suggested that DEX could reduce the levels of TNF-*α*, IL-1β, and IL-10 after sepsis induction at the early stage. The trend of IL-6 detection result was different from the above-mentioned inflammatory cytokines. IL-6 increased at 6–12 h in both the CLP and CLP + DEX groups, and there was no statistical difference between the two groups. A significant decrease was observed in the CLP + DEX group at 24 h, while IL-6 was still at a high level in the CLP group (*p* < 0.05).

### Dexmedetomidine-Mediated Suppression of Inflammation Correlated With Autophagy Levels

Dexmedetomidine was reported to benefit septic challenge by inducing autophagy. To determine whether the DEX-mediated reduction in inflammation in mice is related to autophagy, we determined the levels of autophagy-related proteins using western blotting at different time points. First, we detected the content of LC3II and Beclin-1 in liver tissue of mice. LC3II is a marker of autophagy formation, and the ratio of LC3II to one of the housekeeping proteins is related to the number of autophagosomes (Yoshii and Mizushima, 2017).

**FIGURE 3:**
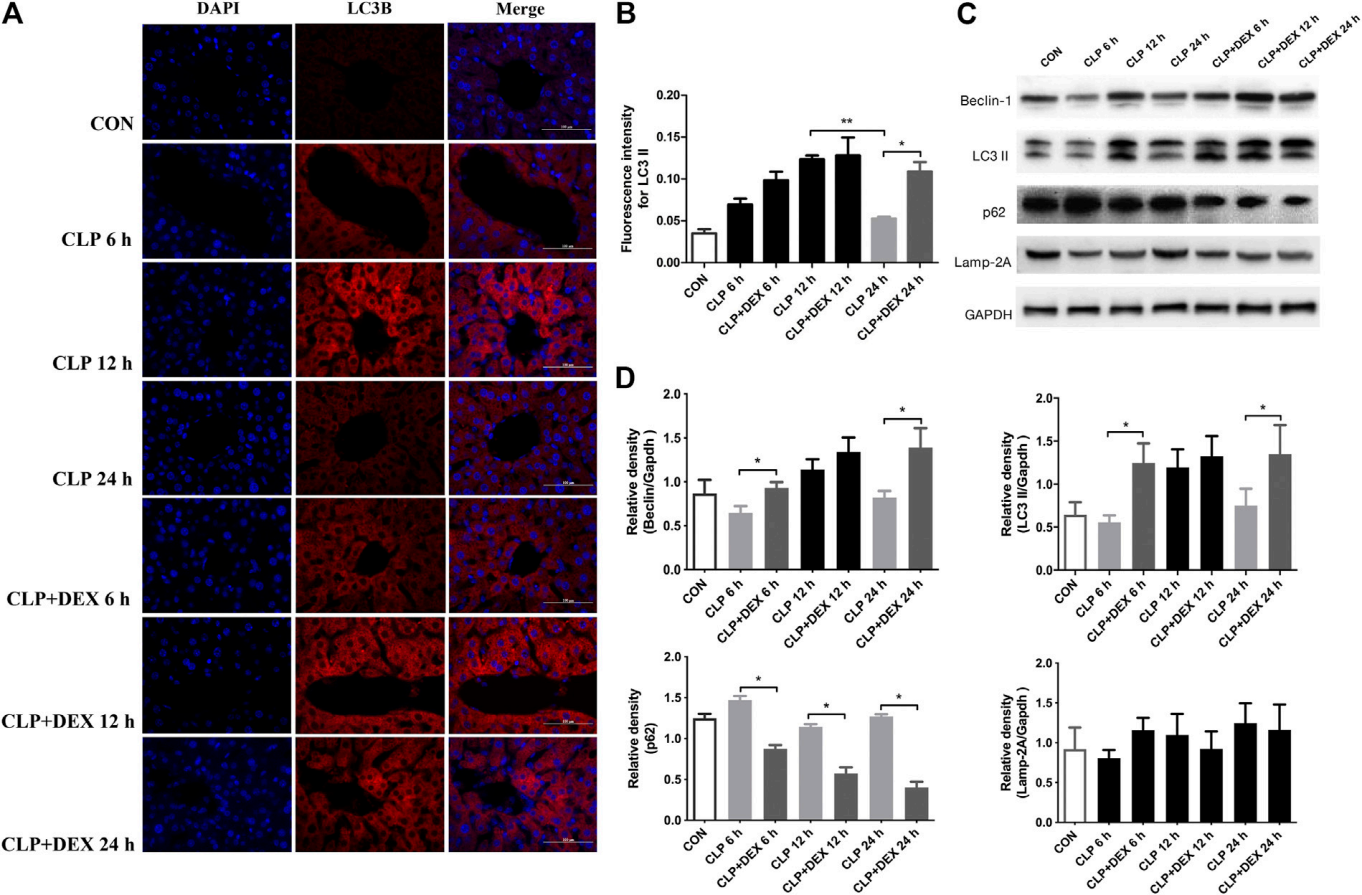
Effect of DEX on the autophagic activity in the livers of mice after CLP surgery. **(A)** The positive expression of LC3II in liver tissue, as detected using immunofluorescence staining (magnification: × 400). **(B)** Analysis of the immunofluorescence intensity for LC3II from **(A)**. **(C)** The protein expression of Beclin-1, LC3II, p62, and LAMP-2 in liver tissues, as determined using western blotting. **(D)** Densitometric analysis of Beclin-1, LC3II, p62, and LAMP-2 from **(C)**. Error bars on the bar graphs represent the mean ± SD. *p < 0.05.

**FIGURE 4:**
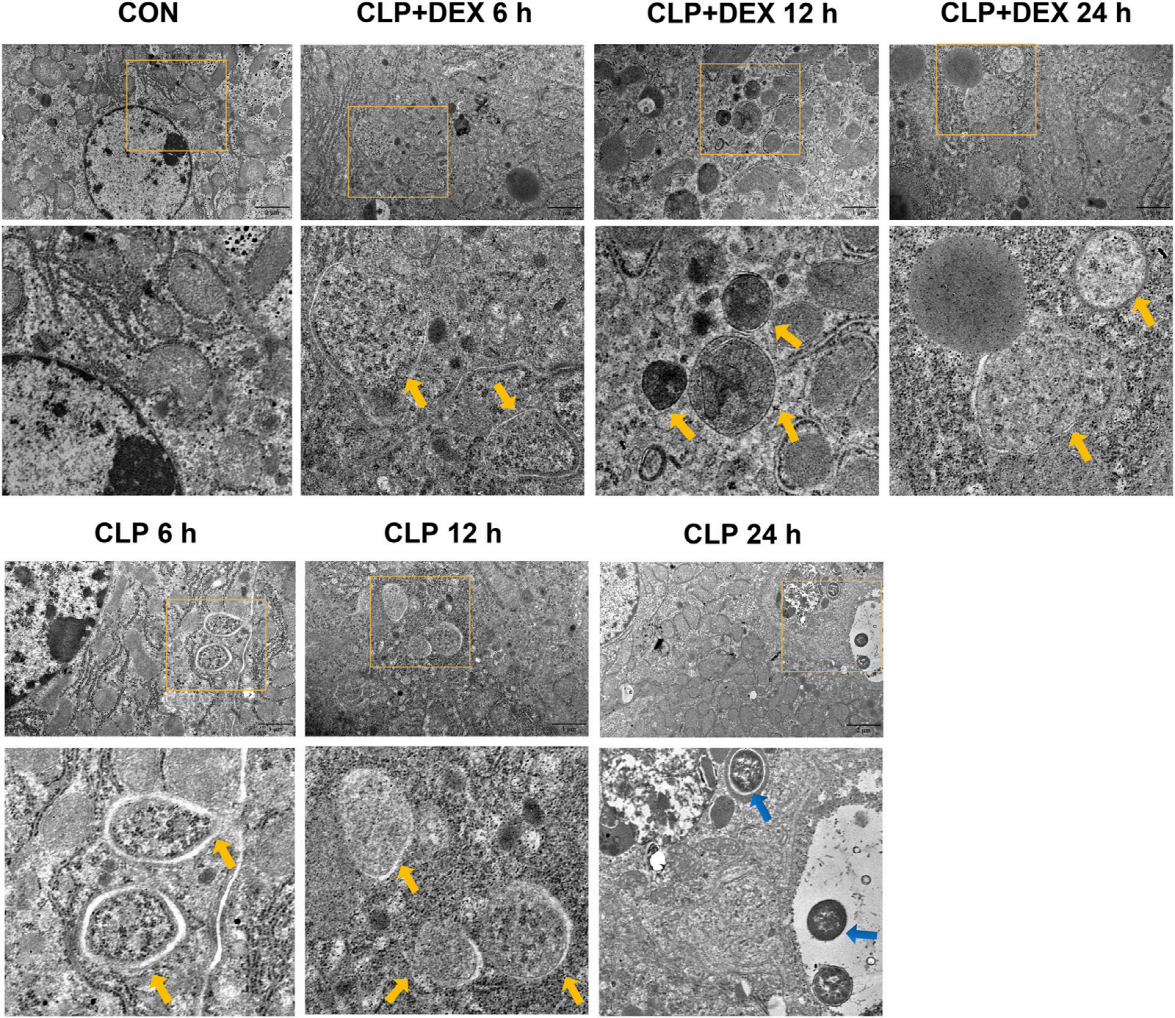
Effect of DEX on the ultrastructure of liver tissues in CLP-induced mice. Autophagosomes in the liver cells are marked by yellow arrows; bacteria in the liver are marked by blue arrows.

Beclin-1 is a key molecule that controls autophagic activity. As shown in Figure 3C, LC3II and Beclin-1 levels in the liver tissues of mice in the CLP group decreased at 6 h post-sepsis, peaked at 12 h, and then decreased at 24 h. In comparison to those in the CLP + DEX group, the LC3II and Beclin-1 levels were lower in the CLP group at 6 and 24 h, while no significant difference was observed at 12 h. Moreover, immunofluorescence analysis of LC3II indicated consistent levels with the western blot data (Figures 3A,B). There were more positive signals of LC3II in the CLP group at 12 h, while the positive signals in the CLP + DEX group were enhanced from 12 to 24 h. Next, we detected the levels of p62. Increased LC3II accompanied by decreased p62 levels is a hallmark of upregulated autophagy (Mizushima, 2004). Our results showed that at each time point before 24 h post-sepsis, the content of p62 in the CLP group was significantly higher than that in the CLP + DEX group. These results strongly suggest that DEX enhanced autophagy. Disruption of the fusion process between autophagosomes and lysosomes, rather than truly enhanced autophagy flux, can lead to LC3II accumulation Settembre et al. (2008); therefore, we tested the level of LAMP-2, which is an important regulator in successful maturation of both autophagosomes and phagosomes (Saftig et al., 2008). We found that the level of LAMP-2 was not significantly different between the groups after sepsis induction. This indicated that the complete autophagic flux was not blocked 24 h after sepsis induction. The increase of autophagosomes at a specific time point was not caused by inhibition of the fusion of autophagosomes and lysosomes. Furthermore, we used transmission electron microscopy (TEM) in liver tissues to observe autophagosomes and autophagosome–lysosomes. The autophagosome structure was clearly observed at different time points in the CLP + DEX group. In the CLP group, typical autophagosome structures were observed in the hepatocytes at 6 and 12 h, but no obvious autophagosomes were found at the 24 h. Scattered bacteria could be seen in the cells at 24 h (Figure 4). Taken together, these *in vivo* results indicated that autophagy of the liver decreased transiently at 6 h, peaked at 12 h, and then declined at 24 h in septic mice. In mice treated with DEX, autophagy activity increased during the earliest stage after CLP modeling and continued for 24 h.

### Dexmedetomidine Induced Autophagy by Upregulating the Sirtuin1/Adenosine Monophosphate-Activated Protein Kinase Signaling Pathway

**FIGURE 5:**
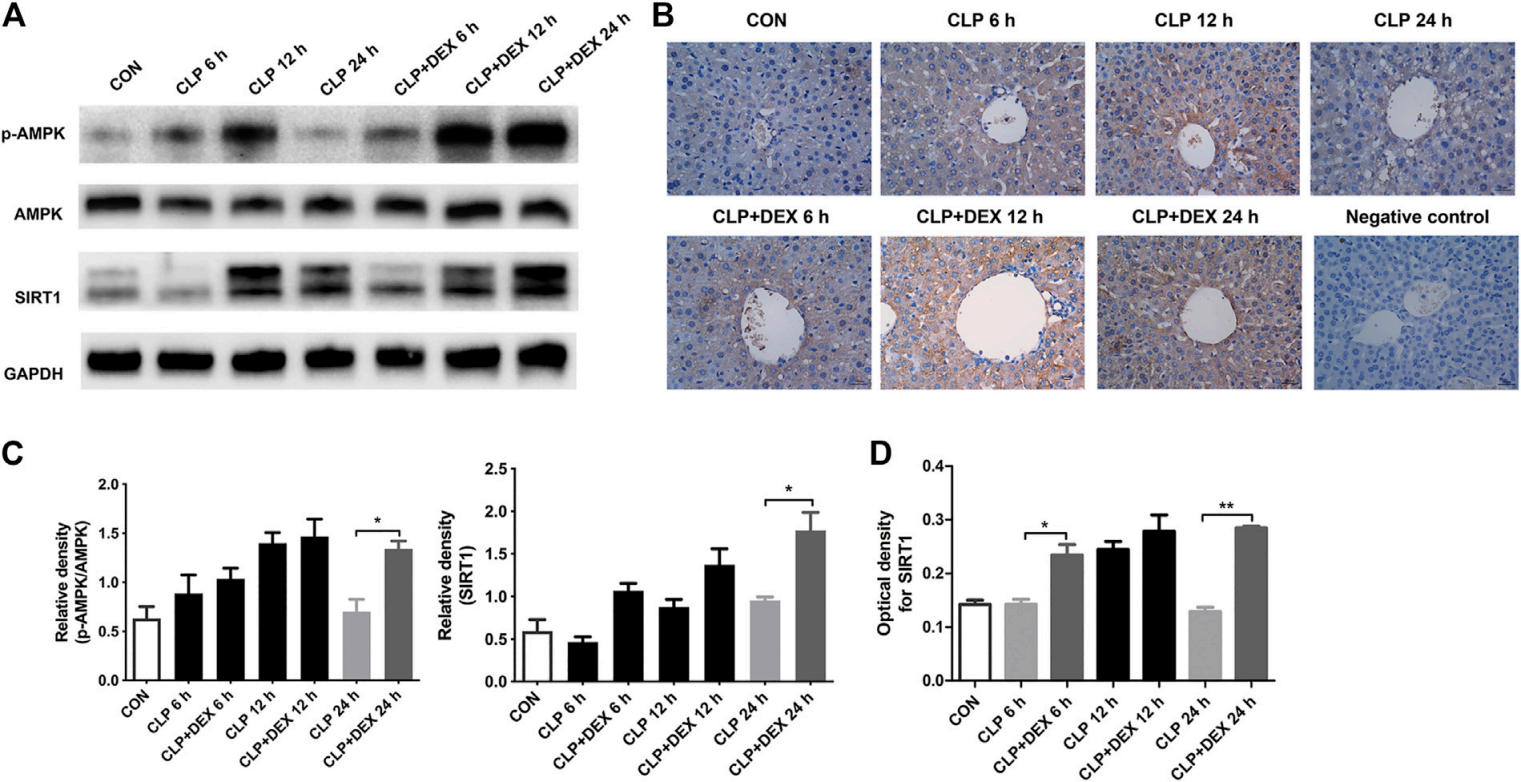
Effect of DEX on AMPK/SIRT1 pathway activation *in vivo*. **(A)** The protein expression of AMPK, *p*-AMPK, and SIRT1 in liver tissues as determined using western blotting. **(B)** The SIRT1 translocation of each group was analyzed using immunohistochemistry (magnification 400 ×). **(C)** Densitometric analysis of the related bands from **(A)**. **(D)** Analysis of the optical densities for SIRT1 from **(B)**. Error bars on the bar graphs represent the mean ± SD. *p < 0.05, **p < 0.01.

The SIRT1/AMPK pathway is a key survival-signaling pathway involved in modulating autophagy. We found that levels of phosphorylated AMPK increased at 12 h and declined at 24 h post-CLP. By contrast, phosphorylated AMPK levels were significantly increased in the CLP + DEX group at 12–24 h. The levels of SIRT1 in septic mice treated with DEX increased significantly at 24 h (Figures 5A,C). Immunohistochemistry results showed that SIRT1 levels in the CLP group increased at 12 h; whereas, SIRT1 levels in the CLP + DEX group were no different from those in the CLP group at 12 h, and SIRT1 levels at 6 and 24 h were higher than those in the CLP group (Figures 5B,D). Based on the previous expression analysis of autophagy-related proteins, we speculated that DEX participated in autophagy progression by regulating the SIRT1/AMPK-signaling pathway.

### Dexmedetomidine Treatment Improved the Viability of D-GalN/LPS-Treated L-02 Cells

The role of autophagy has been investigated in the D-GalN/LPS model of acute liver injury *in vivo* and *in vitro* (Ding, 2014; Liu et al., 2018). As shown in Supplementary Figure S1, cell viability decreased significantly when the treatment concentration of D-GalN/LPS was equal to or more than 20 mM. In the presence of 10 mM D-GalN, the cell viability demonstrated a moderate decrease. Therefore, based on previous studies and our pre-experiments, we treated the L-02 cells with D-GalN at a final concentration of 10 mM combined with 10 ng/ml LPS to establish a hepatocyte injury model *in vitro* for subsequent experiments. Our *in vitro* model showed that 1 and 10 μM DEX significantly improved the viability of L-02 cells treated with D-GalN/LPS.

### Dexmedetomidine Decreased ROS Production and EX527 Reversed the Protective Effect of Dexmedetomidine on DGalN/LPS-Treated L-02 Cells

**FIGURE 6:**
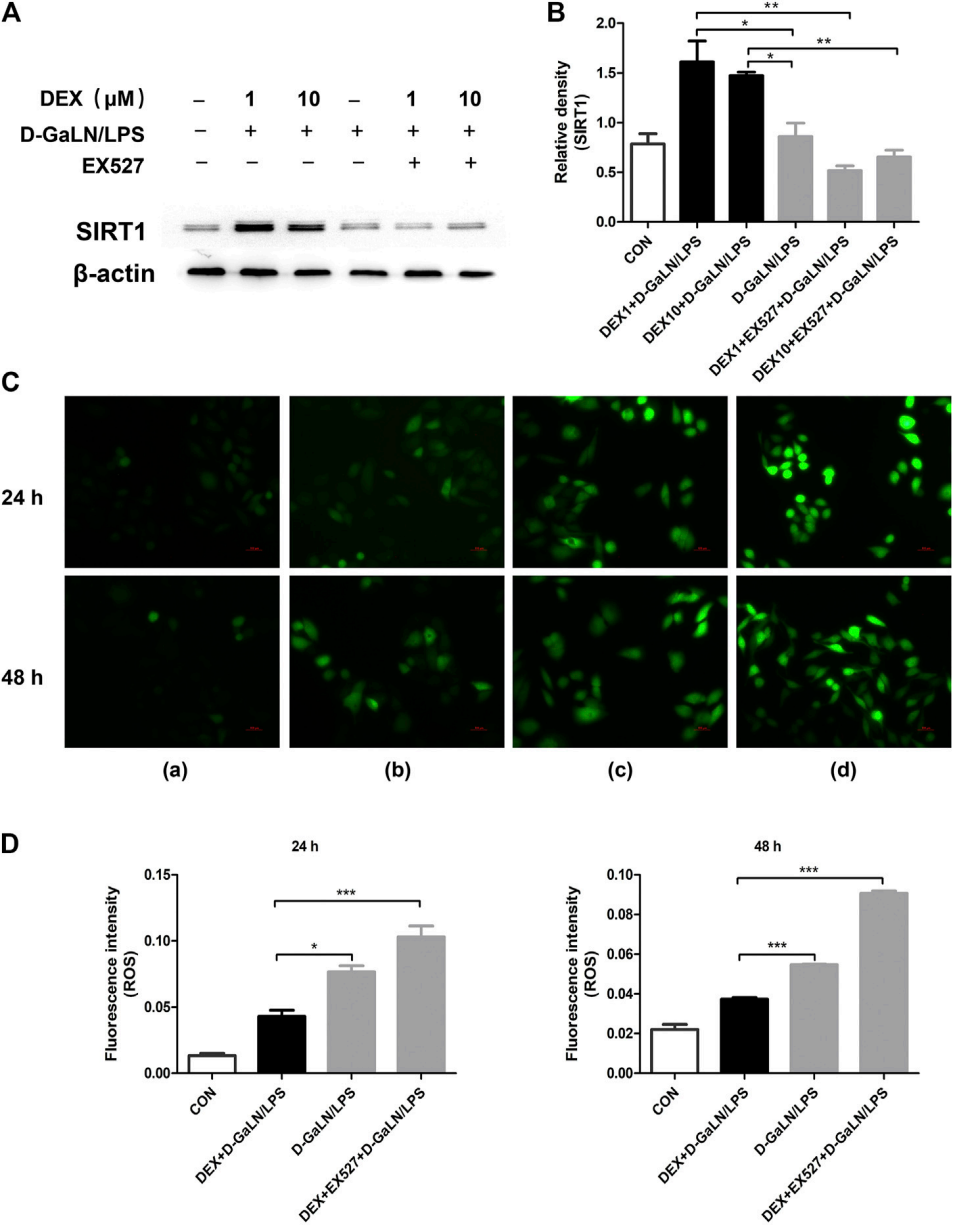
DEX reduced D-GalN/LPS-induced ROS production could reversed by EX527. **(A)** The protein expression of SIRT1 in L-02 cells, as determined using western blotting. **(B)** Densitometric analysis of SIRT1 from **(A)**. **(C)** Intracellular ROS level determination. **(C)** and the quantitative results **(D)**. (A): Control group; (B): DEX + D-GalN/LPS group (DEX: 1 μM, D-GalN: 10 mM, LPS: 10 ng/ml); (C): D-GalN/LPS group (D-GalN: 10 mM, LPS: 10 ng/ml); (D): DEX + EX527+D-GalN/LPS group (DEX: 1 μM, EX527: 10 μM, D-GalN: 10 mM, LPS: 10 ng/ml). Error bars on the bar graphs represent the mean ± SD. *p < 0.05, **p < 0.01, ***p < 0.001.

*In vivo*, we observed that SIRT1 seems to play a regulatory role in DEX-induced autophagy. To explore the specific role of SIRT1 in DEX-induced autophagy, we conducted *in vitro* experiments with SIRT1 inhibitors. To determine whether SIRT1 was mostly inhibited by EX527 in L-02 cells co-cultured with DEX and D-GalN/LPS, we used western blotting to assess SIRT1 levels. As expected, western blotting showed that EX527 (10 μM) significantly downregulated SIRT1 levels in L-02 cells co-cultured with DEX (1, 10 μM) and D-GalN/LPS (Figures 6A,B). The intracellular ROS contents are presented in Figure 6C. As shown in Figure 6D, the ROS content of the DEX + D-GalN/LPS group decreased compared with that of the D-GalN/LPS group and DEX + EX527+D-GalN/LPS group. This revealed that DEX could not reduce ROS induced by D-GalN/LPS in the L-02 cells after SIRT1 was inhibited. Thus, SIRT1 seems to play an important role in the DEX-induced reduction of ROS in D-GalN/LPS-treated L-02 cells.

### Sirtuin1 Is Required for Dexmedetomidine-Induced Autophagy in D-GalN/LPS-Treated L-02 Cells

**FIGURE 7:**
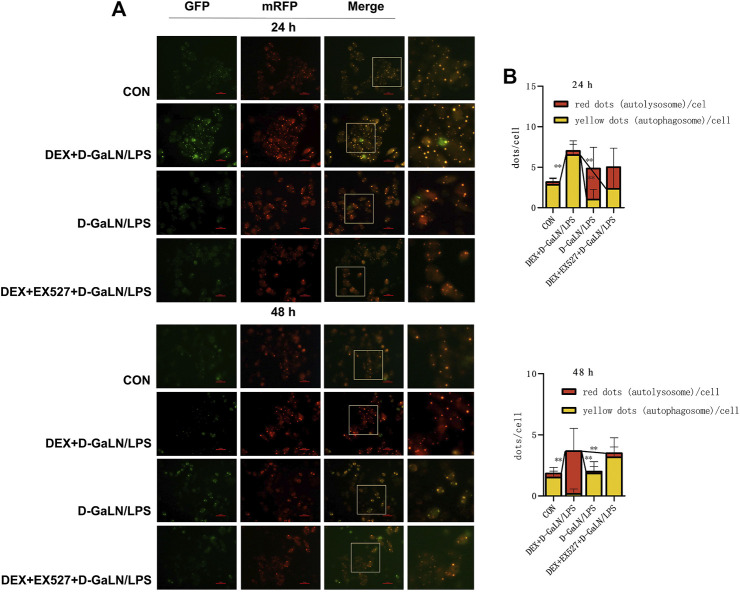
EX527 inhibited DEX-induced autophagy flux in D-GalN/LPS-treated L-02 hepatocytes. **(A)** Fluorescence images of the mRFP-GFP-LC3 in L-02 cells showing the autophagy flux at the indicated time points. **(B)** Mean number of autophagosomes (yellow puncta in the merged images) and autolysosomes (red puncta in the merged images) per cell. Error bars on the bar graphs represent the mean ± SD. **p < 0.01.

We next transfected the cells with mRFP-GFP-LC3 adenovirus expressing a double-labeled fluorescent protein to observed autophagy flux in D-GalN/LPS-treated L-02 cells. After transduction, autophagosomes in cells are shown as yellow dots (the combination of red and green fluorescence), and autolysosomes are shown as red dots because of the extinction of GFP in the acid environment of lysosomes. After co-culturing for 24 h, we found that the D-GalN/LPS group decreased the accumulation of autophagosomes, but increased the formation of autolysosomes; the DEX + D-GalN/LPS group enhanced the formation of yellow autophagosomes in the cytoplasm of D-GalN/LPS-treated L-02 cells; the DEX + EX527+D-GalN/LPS group showed same trends as D-GalN/LPS group, with more red autolysosome in the cytoplasm of L-02 cells (Figures 7A,B). This may indicate that the cells in the D-GalN/LPS group and the DEX + EX527+D-GalN/LPS group were probably in the late stage of autophagy at 24 h, because more lysosomes were observed. However, more yellow autophagosomes could be observed in the DEX + D-GalN/LPS group, which might be in the early stage of autophagy activation. After 48 h, red autolysosomes were obviously distributed in the DEX + D-GalN/LPS group (Figures 7A,B), indicating that DEX promoted a high level of autophagic flux for the conversion from autophagosomes to autolysosomes. By contrast, a small amount of yellow fluorescence and scattered red fluorescence were observed in the D-GalN/LPS group and DEX + EX527+D-GalN/LPS group (Figures 7A,B). Collectively, these results indicated that EX527 obviously impaired the DEX-induced autophagy flux in D-GalN/LPS treated L-02 cells.

## Discussion

The present study found that DEX reduced liver injury in CLP-induced sepsis in mice and significantly reduced their mortality at 24 h. Additionally, we found that the level of autophagy in the liver of CLP-induced sepsis mice peaked at 12 h and decreased significantly at 24 h, whereas in the mice treated with DEX, autophagy was increased at 24 h. An *in vitro* experiment, suggested that the SIRT1 inhibitor EX527 reversed the protective effects of DEX by inhibiting autophagy, and aggravating the production of ROS in D-GalN/LPS treated L-02 cells. Thus, DEX might alleviate the inflammatory responses of liver injury by regulating the SIRT1/AMPK pathway, which might contribute to enhancing autophagy flux.

Several clinical studies have demonstrated that DEX reduces mortality in patients with sepsis (Pandharipande et al., 2010; Zhang et al., 2019). Kawazoe et al. reported an 8% reduction in 28 days mortality in patients with sepsis treated with DEX compare with other sedatives (Kawazoe et al., 2017). Another clinical trial showed a point estimate of 8% reduction in mortality at 14  days in patients treated with DEX (Ohta et al., 2020). Although the results of those two trials did not reach the level of statistical significance, a decreasing trend in mortality after DEX treatment was observed in patients with sepsis, thus physicians might consider the difference in mortality to be clinically significant. Notably, in one clinical study, DEX reduced the risk of death more significantly for patients with sepsis than patients without sepsis (Pandharipande et al., 2010). In other words, the benefit of dexmedetomidine sedation was greater in patients with sepsis. Clinical studies might need more samples to verify the effect of DEX on the survival of patients with sepsis; however, the evidence of DEX’s benefit for the survival of septic model animals is very clear (Hofer et al., 2009; Dardalas et al., 2019; Flanders et al., 2019). In the present study, DEX improved the survival rate of mice significantly in the first 48 h; especially at the 24 h point, at which the survival rate increased by nearly 20%.

We observed that DEX increased the 24 h survival rate of septic mice significantly; therefore, we measured the changes in inflammatory cytokines in mice after 24 h of sepsis. The results showed that at 6 h after sepsis, except for IL-6, TNF-*α*, IL-1β, and IL-10 levels decreased in DEX-treated mice. Interestingly, there was no significant difference in inflammatory factors between the two groups at 12 h. At 24 h, a significant decrease in inflammatory cytokines was observed in the septic mice treated with DEX.

Sepsis was thought to involve the sudden overwhelming production of pro-inflammatory cytokines, leading to tissue edema and multiple organ injury. Autophagy interacts with these inflammatory pathways in response to sepsis (Ho et al., 2016). In the liver, autophagy is important to maintain the balance of energy and nutrients in cell functions, to clear damaged proteins, and resist oxidative stress (Wang et al., 2010). Therefore, disturbance of autophagy in the liver might have a significant impact on liver physiology and diseases (Yin et al., 2008; Takamura et al., 2011). Emerging evidence confirms that autophagy in the liver is induced during the initial 4 h after CLP and declines after this point until 24 h (Chien et al., 2011; Takahashi et al., 2013; Lin et al., 2014). Most of these studies showed that the peak of hepatic autophagy intensity occurs at 6 h. In our study, the peak of autophagy activity in the septic mice appeared 12 h after CLP surgery, and the 24 h decrease in autophagy activity was consistent with the previously reported results. The inconsistency in the peak time of autophagy might reflect the various time-points monitored by different studies. The previous study did not use 12 h as a detection point. The initial increase in autophagy might reflect the early response of the host to oxidative stress and mitochondrial damage caused by sepsis, and might be beneficial in the early stage of sepsis (Chien et al., 2011). The results of the present study showed that the liver function indicators and the expression of inflammatory cytokines decreased in the septic mice at 12 h after surgery. Our results showed that loss or decrease of autophagy exaggerates hepatocyte dysfunction, stimulates expression of inflammatory cytokines, and partially explained the nearly 40% of mouse deaths. However, the reason why autophagy declines to a basal level from 12 to 24 h after CLP remains unclear.

To date, more clinical and animal studies have focused on the anti-inflammatory and organ protective effects of DEX (Li et al., 2015; Zhang et al., 2015; Kong et al., 2017; Zhou et al., 2017; Flanders et al., 2019). The protective mechanism of DEX is being actively explored in many studies. In our study, compared with that in the CLP group, we observed that liver dysfunction (increased serum AST and ALT levels) was highly improved at 24 h after surgery in the CLP + DEX group. Meanwhile, the LC3II ratios and Beclin1 levels in the livers of the CLP mice treated with DEX were higher than those in livers of the CLP mice, whereas the level of p62 was lower, suggesting increased and fluent autophagic flux in the CLP + DEX group. These findings are supported by recent reports of increased autophagy in the heart and kidney of CLP-induced animals treated with DEX (Yu et al., 2019; Yang et al., 2020; Zhao et al., 2020).

**FIGURE 8:**
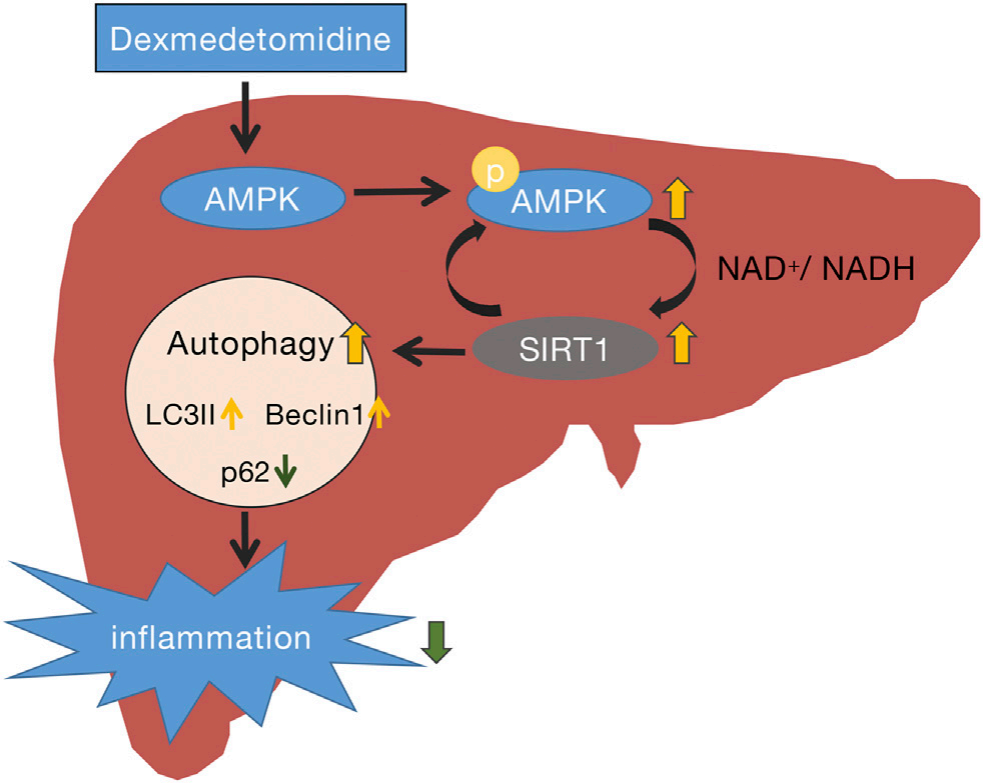
Schematic representation of the potential protective mechanism of dexmedetomidine in liver injury induced by CLP through the AMPK/SIRT1/autophagy pathway.

SIRT1 is an important member of the Sir2 family that plays a key role in preventing liver metabolic damage by regulating autophagy (Song et al., 2015). SIRT1 is regulated by a mixture of interactions with multiple targets, in which kinase AMPK plays an important role. In response to energy depletion or stress conditions, phosphorylated AMPK (*p*-AMPK) increases SIRT1 expression, thereby triggering autophagy to regulate energy homeostasis and metabolic stress (Sun et al., 2018). Recently, DEX has been confirmed to exert its protective effects by activating AMPK and suppressing inflammatory responses (Wang et al., 2018; Yang et al., 2020). A previous study demonstrated that hepatocyte AMPKα1 is a key regulator of liver metabolic and innate immune function (Kikuchi et al., 2020). In our study, we first observed enhanced autophagy activity in liver tissues of septic mice treated with DEX, especially at 24 h after sepsis, and then detected an increase of *p*-AMPK/AMPK and SIRT1 protein levels in the liver of mice with CLP-induced sepsis treated with DEX. This was consistent with a previous study that indicated that DEX increased the levels of *p*-AMPK and *p*-SIRT1 significantly in lung tissues of rats in a model of sepsis-induced lung injury (Wang et al., 2020). Our results further verified that the upregulation of AMPK/SIRT1 levels parallels autophagy levels and shows the opposite trend to the expression of inflammatory cytokines. The present study provides evidence that the organ protection induced by DEX during sepsis may be achieved by influencing autophagy flux (Figure 8).

There are some limitations to the present study. First, AMPK/SIRT1 signaling pathway inhibitors or their components were not used *in vivo* in this study. Thus, the detailed molecular mechanisms *in vivo* need to be further studied. Furthermore, the clinical treatment of sepsis is a comprehensive process and the role of sedation (such as that offered by DEX) under comprehensive treatments has not been considered.

In conclusion, the results of the present study suggest that DEX ameliorates CLP-induced liver injury to some extent by enhancing autophagy. These data also suggest that regulating autophagy levels could be a promising strategy to reduce the medical burden associated with sepsis.

## Data Availability

The original contributions presented in the study are included in the article/Supplementary Material further inquiries can be directed to the corresponding author.
