# Supplementary figures and images for: Dexmedetomidine Protects Against Septic Liver Injury by Enhancing Autophagy Through Activation of the AMPK/SIRT1 Signaling Pathway
Source: Front Pharmacol. 2021 Apr 26;12:658677. doi: 10.3389/fphar.2021.658677 (PMC8109052; doi:10.3389/fphar.2021.658677)

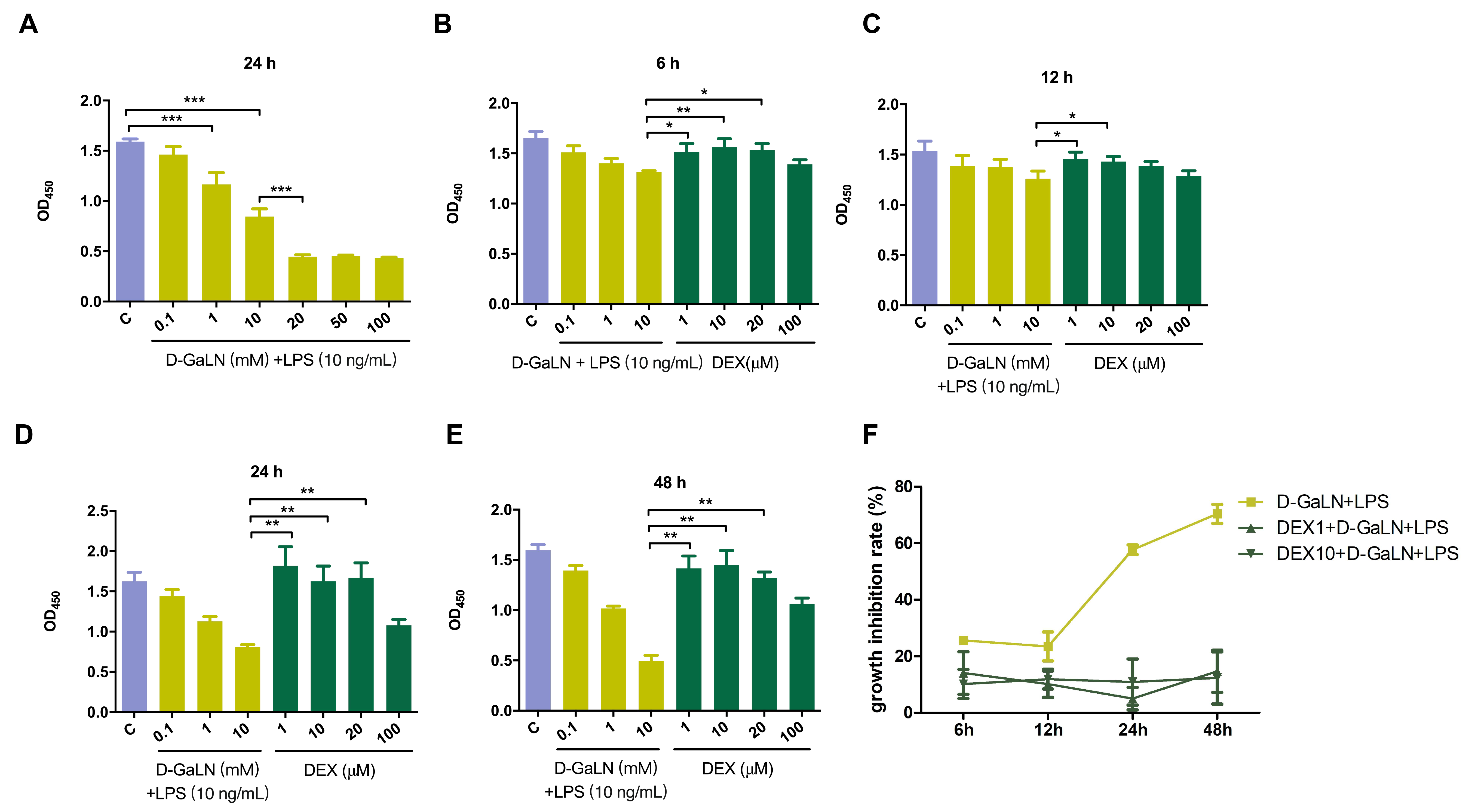

Supplement: Supplementary file 1 [file Image1.TIF]
